# Grape Seed Proanthocyanidins Mitigate the Disturbances Caused by an Abrupt Photoperiod Change in Healthy and Obese Rats

**DOI:** 10.3390/nu14091834

**Published:** 2022-04-27

**Authors:** Jorge R. Soliz-Rueda, Raúl López-Fernández-Sobrino, Francisca Isabel Bravo, Gerard Aragonès, Manuel Suarez, Begoña Muguerza

**Affiliations:** Nutrigenomics Research Group, Departament de Bioquímica i Biotecnologia, Universitat Rovira i Virgili, 43007 Tarragona, Spain; jorgericardo.soliz@urv.cat (J.R.S.-R.); raul.lopez@urv.cat (R.L.-F.-S.); franciscaisabel.bravo@urv.cat (F.I.B.); gerard.aragones@urv.cat (G.A.); begona.muguerza@urv.cat (B.M.)

**Keywords:** cafeteria diet, chrono nutrition, circadian rhythms, phenolic compounds, seasonal rhythms, zeitgebers

## Abstract

Variations in the light/dark cycle and obesogenic diets trigger physiological and behavioral disorders. Proanthocyanidins, in addition to their healthy properties, have recently demonstrated a modulating effect on biological rhythms. Therefore, the aim of this study was to evaluate the administration of a grape seed proanthocyanidin-rich extract (GSPE) to mitigate the disruption caused by a sudden photoperiod change in healthy and cafeteria (CAF)-diet obese rats. For this, 48 photoperiod-sensitive Fischer 344 rats were fed standard or CAF diets for 6 weeks under a standard (12 h light/day, L12) conditions. Then, rats were switched to a long (18 h light/day, L18) or short (6 h light/day, L6) photoperiod and administered vehicle or GSPE (25 mg/kg) for 1 week. Body weight (BW) and food intake (FI) were recorded weekly. Animal activity and serum hormone concentrations were studied before and after the photoperiod change. Hormone levels were measured both at 3 h (ZT3) and 15 h (ZT15) after the onset of light. Results showed the impact of the CAF diet and photoperiod on the BW, FI, activity, and hormonal status of the animals. GSPE administration resulted in an attenuation of the changes produced by the photoperiod disruption. Specifically, GSPE in L6 CAF-fed rats reduced serum corticosterone concentration, restoring its circadian rhythm, increased the T3-to-T4 ratio, and increased light phase activity, while under L18, it decreased BW and testosterone concentration and increased the animal activity. These results suggest that GSPE may contribute to the adaptation to the new photoperiods. However, further studies are needed to elucidate the metabolic pathways and processes involved in these events.

## 1. Introduction

Biological rhythms play an important role in the physiological and metabolic adaptation of the organism to external variations such as the rotation of the Earth around its axis or its translation around the Sun. This adaptation to the time of day (circadian rhythm) or year (seasonal rhythm) allows the optimization of metabolism and energy expenditure (EE) [1,2]. Processes such as cardiovascular activity, endocrine system, blood pressure, body temperature, sleep-wake cycle, kidney activity, gastrointestinal tract, and liver metabolism are regulated to a greater or lesser extent by the circadian rhythm, whose period is usually around 24 h [3,4]. The daylight hours during these 24 h also regulate these processes and allow the organisms to have a reference of the time of the year in which they found themselves. The control center of circadian rhythm is located in the hypothalamic suprachiasmatic nucleus (SCN), and it can maintain the synchronization of the rest of the organism with the external environment via the retina, through the retinohypothalamic tract (RHT) [2,5]. The central master clock can function autonomously, but it can be also reset by external signals or modulators called *zeitgebers* such as light, which plays a crucial role in reprogramming or altering the circadian rhythm [2,6]. In this regard, circadian alteration via changes in day length (photoperiod) has been related to disorders in lipid metabolism and colon functionality [7,8]. Changes in behavioral, feeding patterns, and body weight (BW) gain produced by light/dark shifts have also been reported in the photoperiod-sensitive Fischer 344 (F344) rats [9,10].

The central clock sends signals via the autonomic nervous system or circulating humoral factors, including melatonin and cortisol, to the peripheral clocks to maintain rhythmicity and ensure temporally coordinated physiology [5]. These oscillators, which are present in almost all mammalian tissues, maintain circadian rhythms and regulate tissue-specific gene expression and functionality. Peripheral clocks are also regulated by behavioral signals such as physical activity and, most notably, fasting/feeding states [3]. Diet composition is another important *zeitgeber* for these oscillators, and, for instance, circadian disruptions in hepatic metabolites have been reported in rats fed a cafeteria (CAF) diet [11]. The CAF diet consists of highly palatable, energy-dense, and unhealthy human food, and rats fed a CAF diet are considered a robust model of human metabolic syndrome [12]. Alterations of the master clock in the hypothalamus have also been reported in mice fed a high-fat diet, indicating that diet composition can also modify circadian synchronization to light [6,13]. In fact, in an obesogenic context, alterations of clock gene expressions have been reported in liver, adipose tissue, and hypothalamus, as well as the loss of rhythmicity of hormones and nuclear hormone receptors involved in metabolism and energy utilization, such as corticosterone and thyroid stimulating hormone (TSH), and testosterone both in rodents and humans [6,14,15].

Nevertheless, the interaction between metabolic diseases and circadian rhythms is bidirectional so that circadian misalignment has also been identified as a risk factor for developing metabolic disorders [16]. In this regard, the disruption of hepatic circadian rhythms has been related to non-alcoholic fatty liver disease (NAFLD) [17]. In addition, the circadian rhythm disturbance due to modern lifestyles (shift work, artificial light, fast food, eating time, etc.) has been related to the development of metabolic disorders that in the long term, could lead to type 2 diabetes, cardiovascular diseases, overweight and obesity [18,19]. Additionality, light/dark cycle shifts, and a high fat diet alter the cognitive response in F344 rats [20]. In this regard, it has been pointed out that metabolic disorders typical of the metabolic syndrome, together with other comorbidities, such as depression, sleep disturbances, cognitive dysfunction, and steatohepatitis, could form part of what would be known as the “Circadian Syndrome” [21].

Proanthocyanidins (PACs) are a class of polyphenols constituted by polymers of flavanols and its gallate derivatives, whose healthy activities, including beneficial effects on different key aspects of metabolic syndrome, have been extensively investigated [22]. Several molecular mechanisms have been demonstrated to be involved in their effectivity, including epigenetic modifications, which have recently emerged as important mediators of their properties [23]. In addition, different studies have demonstrated that PACs can modulate both central and peripheral biological rhythms in healthy animals under jet-lag conditions, but also in CAF-fed obese rats [24,25,26,27]. In this regard, the interaction of these phenolic compounds with the clock system has been recently pointed out by our group as another mechanism involved in their beneficial effects [28].

Therefore, the aim of the present study was to investigate if PACs can contribute to restoring circadian disruption caused by light/dark cycle disturbances in standard (STD) conditions and diet-induced obesity. For this purpose, STD- and CAF-fed rats were transferred abruptly from STD (12 h light/day, L12) to long (18 h light/day, L18) or short (6 h light/day, L6) photoperiods. BW gain and food intake were recorded weekly and animal activity and the serum hormones levels of T3 and T4, corticosterone, testosterone (as representative hormones of hypothalamic–pituitary–thyroidal, –adrenal and –gonadal axis modulating hormones, respectively) and melatonin were analyzed before and after photoperiod disruption.

## 2. Materials and Methods

### 2.1. Grape Seed Proanthocianidin-Rich Extract

The grape seed proanthocyanidin-rich extract (GSPE) used in this study was provided by Les Dérives Résiniques et Terpéniques (Dax, France). This was obtained from white grape seed. The phenolic profile of this extract is mainly composed of catechin, epicatechin, gallic acid, epicatechin gallate, and dimers, trimers, and tetramers of proanthocyanidins [29].

### 2.2. Animal Experiment Procedure

**Figure 1:**
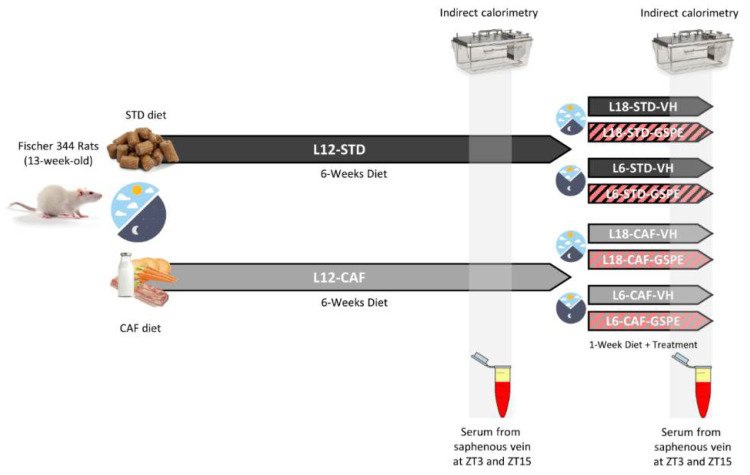
Experimental design to evaluate the effect of GSPE after an abrupt disturbance of the photoperiod. STD- and CAF-fed rats were switched to a new light–dark cycle and VH or GSPE were administered to the animals. STD, Standard diet-fed rats; CAF, Cafeteria diet-fed rats; VH, rats administered vehicle; GSPE, rats administered 25 mg/kg grape seed proanthocyanidin-rich extract; L12, standard photoperiod 12 h light per day; L18, long photoperiod 18 h light per day; L6, short photoperiod 6 h light per day.

The animals used were 13-week-old male F344 rats from Charles Rives Laboratories (Barcelona, Spain) housed under STD laboratory conditions at 22 °C and 12 h light/dark cycle with ad libitum access to food and drinking water. After a two-week acclimation period, rats were weighed and randomly divided into two dietary groups (Figure 1). One group (L12-STD) was fed STD chow diet (2.90 kcal/g; A04, Panlab, Barcelona, Spain), and the other group (L12-CAF), a CAF diet for 6 weeks (*n* = 24/group). After this time, animals were transferred from L12 to a L18 or L6 photoperiods and administered vehicle (VH), which was condensed milk 1/5 diluted, or GSPE (25 mg/kg) dissolved in VH for 1 week more. Thus, the animals were grouped into 8 different groups (*n* = 6). The CAF diet was prepared every day and contained bacon, cookie with paté, cookie with cheese, carrots, ensaïmada (pastry), STD chow, and sweetened milk (22% sucrose *w*/v), and its caloric distribution was 56.43% carbohydrate, 45.72% lipid, and 9.5% protein [30]. The onset of light was at 8:00 a.m. and defined as *zeitgeber* time 0 (ZT0). Both VH and GSPE treatments were administered to the rats at ZT0. BW and food intake (FI) were recorded weekly during the whole experimental procedure. Blood was collected from the saphenous vein in non-heparinized tubes, incubated for 1 h at room temperature, and immediately centrifuged at 1200× *g* for 15 min at 4 °C to collect the serum. To study the light/dark cycle, blood was extracted at two different times, ZT3 and ZT15 (11:00 a.m. and 11:00 p.m.) in the sixth and seventh weeks.

Animal procedures were approved by The Animal Ethics Committee of the University Rovira i Virgili (Tarragona, Spain) and the Generalitat de Catalunya (reference number 9495, 18 September 2019) and were carried out in accordance with Directive 86/609EEC of the Council of the European Union and the procedure established by the Departament d’Agricultura, Ramaderia i Pesca of the Generalitat de Catalunya.

### 2.3. Indirect Calorimetry

Indirect calorimetry was performed on all animals one week before the photoperiod change and two days before sacrifice after the administration of VH or GSPE, using an Oxylet Pro System (Panlab, Barcelona, Spain) and the Metabolism 2.1.02 (Panlab, Cornellà, Spain) software program. Lipid and glucose oxidation were calculated with oxygen consumption (VO_2_) and carbon dioxide production (VCO_2_) values given by the Oxylet LE 405-gas analyzer (PANLAB, Barcelona, Spain). At each time point, the program Metabolism 2.1.02 (PANLAB, Barcelona, Spain) calculated the respiratory quotient (RQ) as the VCO_2_/VO_2_ ratio. Using the stoichiometric equations of Frayn [31] and assuming a nitrogen excretion rate (n) of 135 μg/kg × min [32], we used the formula (g/min) = 4.55 × VCO_2_ − 3.21 × VO_2_ − 2.87 n for calculating the oxidation of carbohydrates and the formula (g/min) = 1.67 × VO_2_ − 1.67 × VCO_2_ − 1.92 n for calculating the oxidation of lipids. To obtain the EE from lipids and carbohydrates, the lipid and carbohydrate rates were multiplied by 37 and 16, respectively, using the Atwater general conversion factor [33]. In addition, indirect calorimetry allows us to measure the vertical activity of the rat with sensors that detect when rats stand up on their two hind legs. In order to study the effects of the photoperiod change and the adaptation to the new light/dark cycle of the animals, the results of the light and darkness phases were separated, taking as reference ZT12, and the measurements were analyzed for 12 h around ZT12, 6 h from ZT6 to ZT12, and 6 h from ZT12 to ZT18.

### 2.4. Hormone Analysis

Serum hormone concentrations were measured by liquid chromatography coupled to a triple quadrupole mass spectrometer (LC-QqQ). Serum samples were thawed at 4 °C and 50 μL of serum were mixed with 250 μL of methanol containing the internal STD (2 ng/mL). Then, the mixture was vortexed and centrifuged for 5 min at 4 °C and 252× *g*. The supernatant was transferred to a new tube and mixed with 700 μL of 0.1% formic acid in water. The sample was loaded to a SPE system previously conditioned with methanol and 0.1% formic acid in water. The cartridge was washed with 0.1% formic acid in water and dried under high vacuum. The compounds were eluted with 500 μL of methanol. Samples were evaporated in a SpeedVac at 45 °C and reconstituted with 50 μL of water:methanol (60:40, *v*/*v*) and transferred to a glass vial for analysis. The hormones detected were melatonin, corticosterone, triiodothyronine (T3), thyroxine (T4), and testosterone. The analytical column was a Zorbax Eclipse C18 (150 × 2.1 mm) from Agilent Technologies.

### 2.5. Statistical Analysis

Data were represented as means ± standard error of mean (S.E.M.) of each group and for this data normality and homogeneity of variance were tested by the Shapiro–Wilk and Levene tests, respectively. Differences between groups were assessed by repeated-measures ANOVA followed by a LSD post-hoc test for BW gain and accumulative food intake, and for the rest of parameters three-way and two-way ANOVA were used, followed by a LSD post-hoc test. In order to study two time points to analyze diurnal/nocturnal cycle, a paired Student’s *t*-test was carried out. A summary of the results is found in Table S1. These statistical analyses were performed using the statistical software package SPSS Statistics 22 (SPSS Inc., Chicago, IL, USA). A *p*-value ≤ 0.05 was considered statistically significant.

## 3. Results

### 3.1. Abrupt Photoperiod Transfer Caused Changes in Body Weight Gain and Food Intake Patterns in CAF-Fed Rats: Effects of Grape Seed Proanthocyanidins

**Figure 2:**
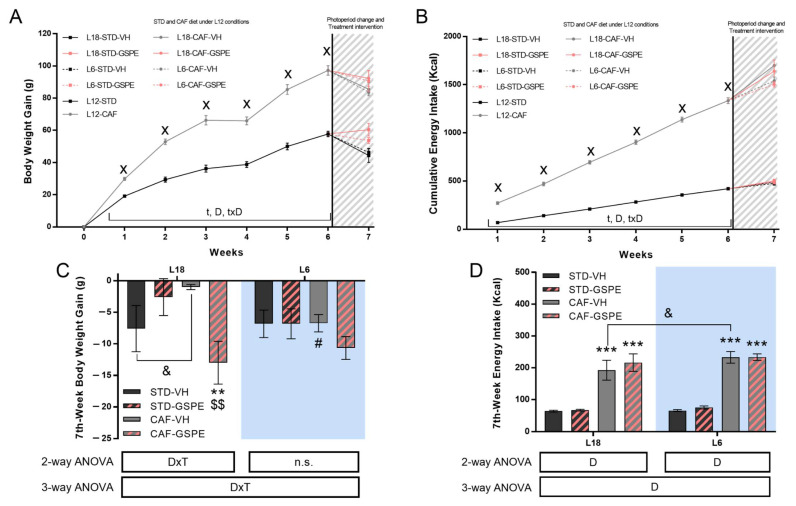
Body weight gain and cumulative food intake. (**A**) Body weight gain and (**B**) Cumulative energy intake over the experiment. (**C**) Body weight gain in the seventh week for L18 and L6 conditions and (**D**) Cumulative energy intake for L18 and L6 conditions (STD-VH, STD-GSPE, CAF-VH, and CAF-GSPE) in the seventh week. Values are expressed as the mean ± S.E.M. (*n* = 24 for L12 groups and *n* = 6 for L18 and L6 groups). x Indicates significant differences using repeated measured-ANOVA followed by a Student’s *t*-test between L12-STD vs. L12-CAF (*p* ≤ 0.001). D, diet effect; DxT, interaction between treatment and Diet; t, time effect; txD, interaction between time and diet effect. n.s., no significant differences. ** or *** Indicate significant differences by diet effect (*p* ≤ 0.01 and *p* ≤ 0.001, respectively), $$ Indicates significant differences by treatment effect (*p* ≤ 0.01), # Indicates significant differences by photoperiod effect using 2-way and 3-way ANOVA followed by a LSD post-hoc test (*p* ≤ 0.05). & Indicates tendency using a LSD post-hoc test (*p* = 0.1–0.051). STD, Standard diet-fed rats; CAF, Cafeteria diet-fed rats; VH, rats administered vehicle; GSPE, rats administered 25 mg/kg grape seed proanthocyanidin-rich extract; L12, standard photoperiod 12 h light per day; L18, long photoperiod 18 h light per day; L6, short photoperiod 6 h light per day. The blue background corresponds to the groups under L6 conditions.

Rats fed a CAF diet showed a significantly increased BW gain compared to rats fed a STD diet (Figure 2A). The increase in BW gain was observed from the first week of CAF administration and during the 6 weeks that the rats were in L12 conditions. According to this, increases in food and energy intake were observed in the CAF-fed group during the 6-week period of the experiment compared to STD-rats (Table 1 and Figure 2B).

After 6 weeks in the L12 photoperiod, the rats were abruptly transferred to the L18 or L6 photoperiods. This change in light/dark cycles caused a loss of BW gain in some of the groups (Figure 2C). Regarding animals transferred to L18, an effect of diet was observed in CAF-fed rats administered VH, since this group tended to lose less BW compared to L18-STD-VH animals. In addition, GSPE administration to CAF-rats resulted in a substantial loss of BW gain compared to CAF-VH rats (*p* = 0.005). Interestingly, a photoperiod effect was observed in the CAF-VH group since this group did not lose BW when transferred to the L18 photoperiod but tended to lose it in L6 conditions (*p* = 0.021). GSPE administration in L6 conditions did not cause any change in BW loss.

The energy intake after the photoperiod change was higher in CAF-fed rats than in STD-rats both in L18 and L6 animals, especially due to the consumption of carbohydrates and fats (Table 1 and Figure 2D). Interestingly, the ingestion of carbohydrates was affected by the photoperiod since CAF-fed rats under L6 conditions ingested more energy from carbohydrates than CAF-fed rats under L18 conditions (*p* = 0.033). GSPE administration promoted a higher energy intake from fat in CAF-fed rats in both L18 and L6 photoperiods (*p* < 0.001 and *p* = 0.018, respectively). Moreover, this effect was different depending on the photoperiod since the increase in energy intake from fat was higher in L18 compared to L6. It was also observed that GSPE administration tended (*p* = 0.064) to increase the energy intake from protein in STD-fed rats in the L6 photoperiod.

### 3.2. Grape Seed Proanthocyanidins Attenuated the Impact of Photoperiod Change on Energetic Expenditure

**Figure 3:**
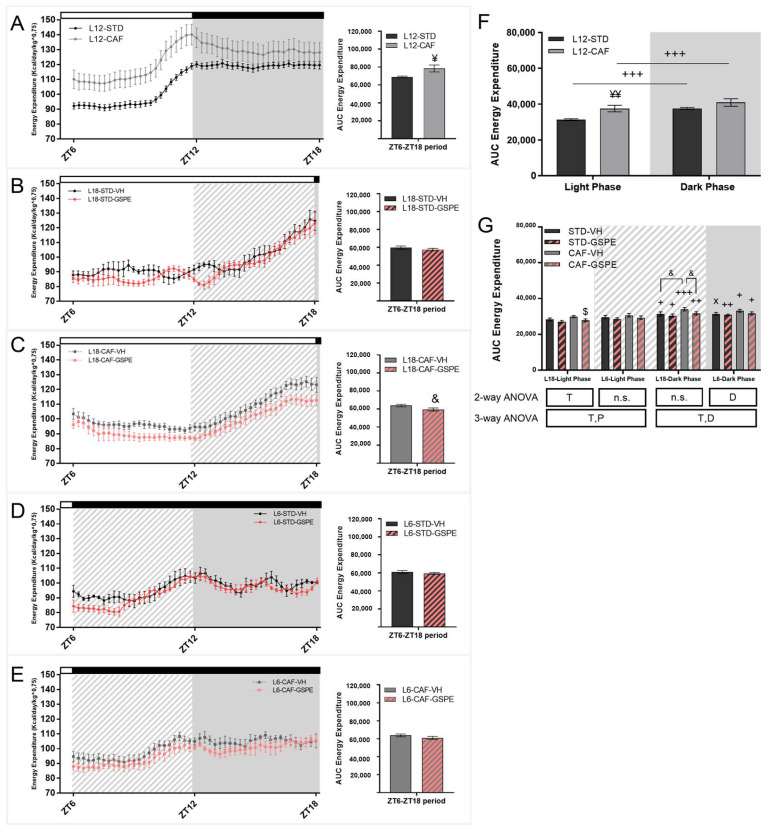
Energy expenditure of STD- and CAF-fed rats in the sixth week of the experiment under L12 conditions and in the seventh week of the experiment under L18 and L6 conditions. (**A**) EE for STD- and CAF-fed rats under the L12 photoperiod and the corresponding AUC. (**B**) EE for STD-fed rats and VH or GSPE administration under the L18 photoperiod and AUC. (**C**) EE for CAF-fed rats administered VH or GSPE under the L18 photoperiod and the corresponding AUC. (**D**) EE for STD-fed rats administered VH or GSPE under the L6 photoperiod and the corresponding AUC. (**E**) EE for CAF-fed rats administered VH or GSPE under the L6 photoperiod and the corresponding AUC. (**F**) AUC of EE for L12 conditions groups. (**G**) AUC of EE for L6 and L18 conditions groups. For L12 conditions values are expressed as the mean ± S.E.M. (*n* = 24). ¥ or ¥¥ Indicate significant differences using an unpaired Student’s *t*-test between L12-STD vs. L12-CAF (*p* ≤ 0.05 and *p* ≤ 0.01, respectively). Values are expressed as the mean ± S.E.M. (*n* = 6). D, diet effect; T, GSPE treatment effect; P, photoperiod effect; n.s., no significant differences. $ Indicates significant differences by treatment effect. & Indicates tendency using a LSD post-hoc test (*p* = 0.1–0.051). +, ++ or +++ Indicate significant differences using a paired Student’s *t*-test between Light Phase vs. Dark Phase for each group (*p* ≤ 0.05, *p* ≤ 0.01 and *p* ≤ 0.001, respectively). x Indicates tendency using a paired Student´s *t*-test between Light Phase vs. Dark Phase for each group (*p* = 0.1–0.051). EE, Energy Expenditure; AUC, Area under curve; STD, Standard diet-fed rats; CAF, Cafeteria diet-fed rats; VH, rats administered vehicle; GSPE, rats administered 25 mg/kg grape seed proanthocyanidin-rich extract; L12, standard photoperiod 12 h light per day; L18, long photoperiod 18 h light per day; L6, short photoperiod 6 h light per day. Grey background, dark phase. slash background, transitions phase.

The EE data obtained over the day in all the groups are shown in Figure 3A–E. Indirect calorimetry data showed differences in EE between CAF- and STD-fed rats in L12 animals, before the photoperiod change. The expenditure was lower in STD-fed diet rats compared to their CAF-fed counterparts (Figure 3A). The increase in EE in CAF animals under L12 conditions disappeared when the photoperiod transfer was performed (Figure 3F, G). A tendency to increase EE (*p* = 0.053) by CAF animals was only found in the L18 photoperiod in the transition phase, a period change from dark in L12 conditions to light in the L18 photoperiod (from ZT12 to ZT18). In this context, GSPE administration to CAF-fed rats in the L18 photoperiod reduced EE during the light phase (*p* = 0.041) and tended to reduce it during the transition phase (*p* = 0.057). Despite this effect, the rhythm set by the light/dark cycle was maintained for both groups. This rhythm was only minimally altered in STD-VH rats in the L6 photoperiod, where the difference between light and dark phases in EE was not significant (*p* = 0.055) while it recovered with GSPE administration (*p* = 0.008).

**Figure 4:**
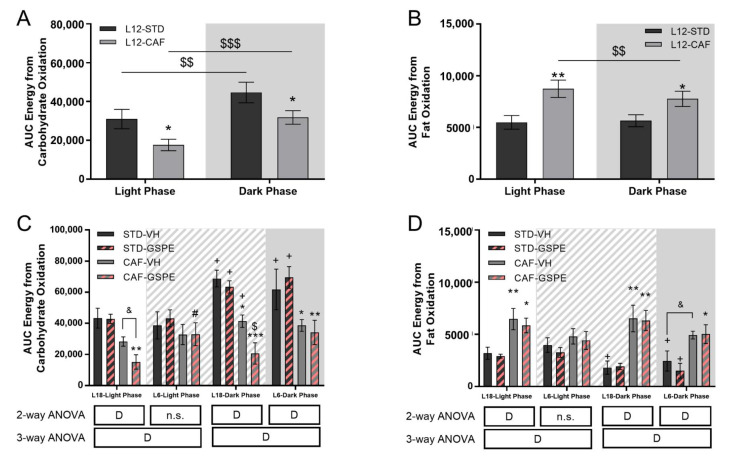
Energy from carbohydrate or fat oxidation of STD- and CAF-fed rats in the sixth week of the experiment under L12 conditions and in the seventh week of the experiment under L18 and L6 conditions. (**A**) AUC of energy from carbohydrate oxidation for STD- and CAF-fed rats under the L12 photoperiod. (**B**) AUC of energy from fat oxidation for STD-fed rats and VH or GSPE administration under the L18 photoperiod. (**C**) AUC of energy from carbohydrate oxidation for L6 and L18 conditions groups. (**D**) AUC of energy from fat oxidation for L6 and L18 conditions groups. For L12 conditions, values are expressed as the mean ± S.E.M. (*n* = 24). Values are expressed as the mean ± S.E.M. (*n* = 6). D, diet effect; n.s., no significant differences. *, ** or *** Indicate significant differences by diet effect (*p* ≤ 0.05, *p* ≤ 0.01 and *p* ≤ 0.001, respectively), $, $$ or $$$ Indicate significant differences by treatment effect (*p* ≤ 0.05, *p* ≤ 0.01 and *p* ≤ 0.001, respectively), # Indicates significant differences by photoperiod effect using 2-way and 3-way ANOVA followed by a LSD post-hoc test (*p* ≤ 0.05). & Indicates tendency using a LSD post-hoc test (*p* = 0.1–0.051). + Indicate significant differences using a paired Student´s *t*-test between Light Phase vs. Dark Phase for each group (*p* ≤ 0.05, respectively). AUC, Area under curve; STD, Standard diet-fed rats; CAF, Cafeteria diet-fed rats; VH, rats administered vehicle; GSPE, rats administered 25 mg/kg grape seed proanthocyanidin-rich extract; L12, standard photoperiod 12 h light per day; L18, long photoperiod 18 h light per day; L6, short photoperiod 6 h light per day. Grey background, dark phase. slash background, transitions phase.

Regarding the energy source used by the animals, the results showed that STD-fed rats preferentially oxidized carbohydrates (Table 1), both in the light and dark phases, although the two groups showed a clear rhythm marked by the light/dark cycle with higher carbohydrate oxidation during the night (Figure 4A). However, CAF animals used mainly fat throughout the day, both in the light and dark periods. Moreover, only the CAF-group showed a clear rhythm marked by the light/dark cycle with higher fat oxidation during the light phase (Figure 4B). The energy from carbohydrate oxidation (Figure 3C and Table 1) tended to decrease with GSPE administration in CAF-fed rats in the L18 photoperiod in the light phase (*p* = 0.070) and significantly decreased in the dark phase (*p* = 0.044). In addition, the photoperiod change promoted a loss of the rhythm marked by the light/dark cycle in CAF-fed rats, which was maintained in STD-fed rats in both photoperiods. Regarding energy from fat oxidation (Figure 4D and Table 1), the higher fat oxidation by CAF rats was generally maintained. The main change was the alteration to the rhythm by CAF-fed rats when they were transferred to L6 and L18. Furthermore, in the L6 photoperiod, no differences were found between STD- and CAF-fed rats during the light phase.

### 3.3. Cafeteria Diet Decreases the Activity of Animals in Standard Conditions and Grape Seed Proanthocyanidins Attenuated Photoperiod Changes in the Activity of Rats

**Figure 5:**
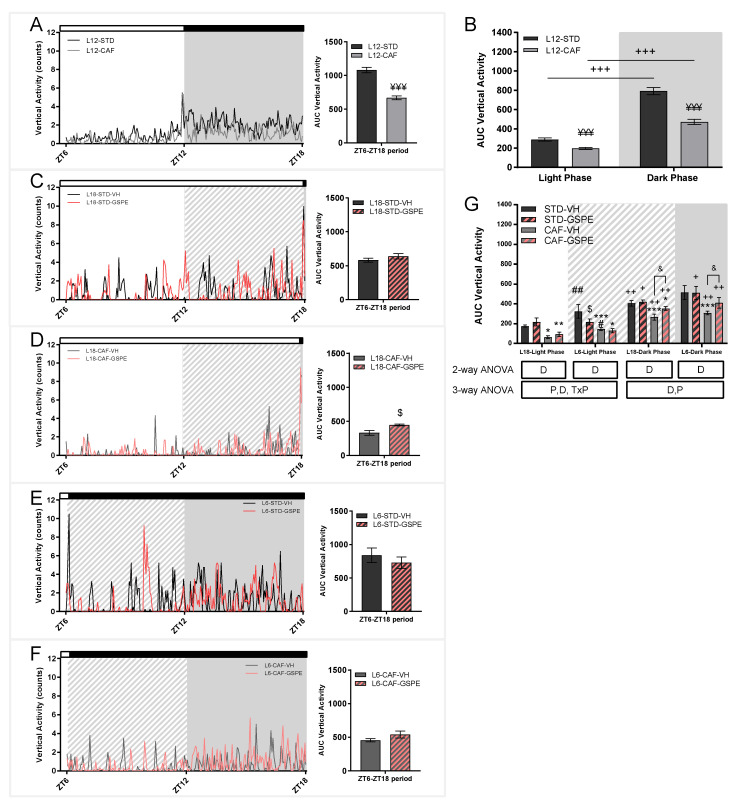
Activity of STD- and CAF-fed rats in the sixth week of the experiment under L12 conditions and in the seventh week of the experiment under L18 and L6 conditions. (**A**) Activity for STD- and CAF-fed rats under the L12 photoperiod and the corresponding AUC. (**B**) Activity for STD-fed rats administered VH or GSPE under the L18 photoperiod and the corresponding AUC. (**C**) Activity for CAF-fed rats administered VH or GSPE under the L18 photoperiod and the corresponding AUC. (**D**) Activity for STD-fed rats administered VH or GSPE under the L6 photoperiod and the corresponding AUC. (**E**) Activity for CAF-fed rats administered VH or GSPE under the L6 photoperiod and the corresponding AUC. (**F**) AUC of activity for L12 conditions groups with light phase and dark phase separated. (**G**) AUC activity for L6 and L18 conditions groups with light phase and dark phase separated. For L12 conditions, values are expressed as the mean ± S.E.M. (*n* = 24). ¥¥¥ Indicates significant differences using an unpaired Student´s *t*-test between L12-STD vs. L12-CAF (*p* ≤ 0.001). Values are expressed as the mean ± S.E.M. (*n* = 6). D, diet effect; P, photoperiod effect; TxP, interaction between treatment and photoperiod. *, ** or *** Indicate significant differences by diet effect (*p* ≤ 0.05, *p* ≤ 0.01 and *p* ≤ 0.001, respectively), $ Indicates significant differences by treatment effect, ## Indicate significant differences by photoperiod effect (*p* ≤ 0.01, respectively) using 2-way and 3-way ANOVA followed by a LSD post-hoc test (*p* ≤ 0.05). & Indicates tendency using a LSD post-hoc test (*p* = 0.1–0.051). +, ++ or +++ Indicate significant differences using a paired Student´s *t*-test between Light Phase vs. Dark Phase for each group (*p* ≤ 0.05, *p* ≤ 0.01 and *p* ≤ 0.001, respectively). AUC, Area under curve; STD, Standard diet-fed rats; CAF, Cafeteria diet-fed rats; VH, rats administered vehicle; GSPE, rats administered 25 mg/kg grape seed proanthocyanidin-rich extract; L12, standard photoperiod 12 h light per day; L18, long photoperiod 18 h light per day; L6, short photoperiod 6 h light per day. Grey background, dark phase. slash background, transitions phase.

A change in the behavioral pattern of the rats could be observed depending on the diet administered since the CAF-fed rats displayed less activity than their STD-fed counterparts (Figure 5A). STD- and CAF-groups displayed less activity in their resting period, light phase, but both in light and dark phases, the activity was decreased in the CAF-fed animals (Figure 5B). The change in the photoperiod also had a major impact on the activity pattern of the rats (Figure 5C–F). A reducing effect on activity because of the CAF diet was observed in both photoperiods and in both phases, light and dark. The activity of the rats was increased when they were transferred to the L6 photoperiod compared to rats that were transferred to the L18 photoperiod, both STD-fed (*p* = 0.002) and CAF-fed rats (*p* = 0.032), during light phase. Interestingly, GSPE administration reduced activity in this phase only in STD-fed rats in the L6 photoperiod (*p* = 0.024). During the dark phase, GSPE administration tended to increase activity in both L6 (*p* = 0.060) and L18 (*p* = 0.051). GSPE-treated and CAF-fed rats increased activity when it was analyzed over the whole day (Figure 5D). Despite these changes, the two CAF-fed groups maintained the rhythm set by the light/dark cycle when the activity was analyzed separately (Figure 5G). Additionally, the STD-fed rats in the L6 photoperiod lost the rhythm set by the light/dark cycle, which recovered with GSPE administration (*p* = 0.014).

### 3.4. GSPE Modulates Rhythm Alterations Caused by Abrupt Photoperiod Change in Serum Hormones

**Figure 6:**
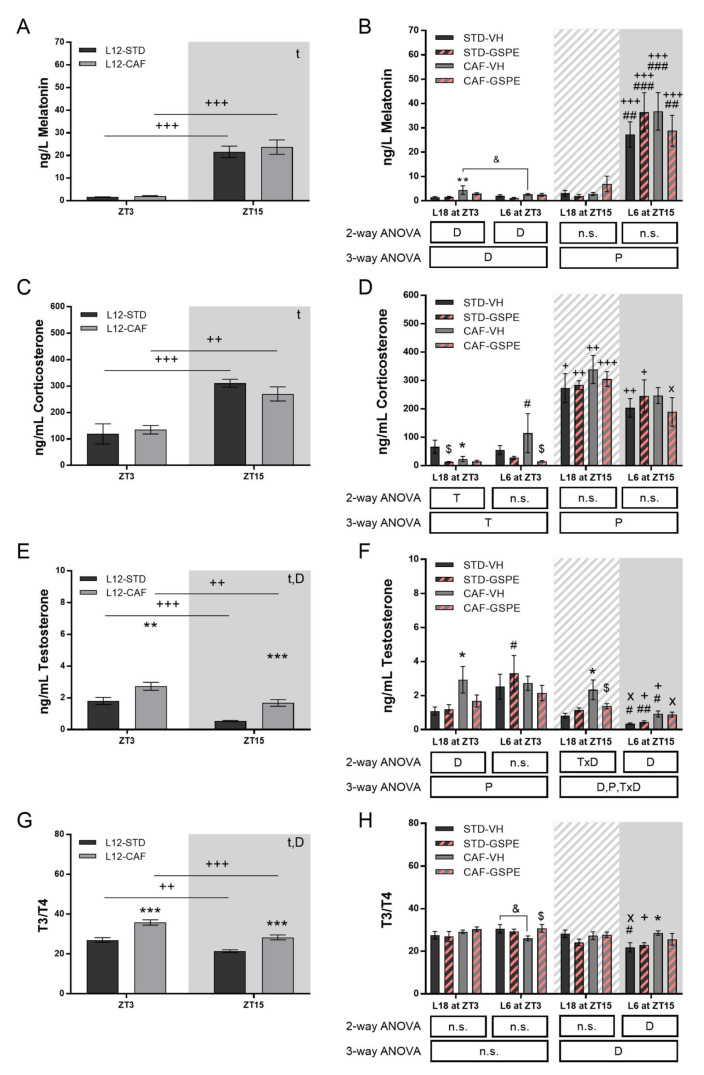
Serum hormones of STD- and CAF-fed rats in the sixth week of the experiment under L12 conditions and in the seventh week of the experiment under L18 and L6 conditions. Serum melatonin at ZT3 and ZT15 (**A**) in L12 and (**B**) L18 and L6 conditions. Serum corticosterone at ZT3 and ZT15 (**C**) in L12 and (**D**) L18 and L6 conditions. Serum testosterone at ZT3 and ZT15 (**E**) in L12 and (**F**) L18 and L6 conditions. Serum T3-to-T4 ratio at ZT3 and ZT15 (**G**) in L12 and (**H**) L18 and L6 conditions. Values are expressed as the mean ± S.E.M. (*n* = 6). D, diet effect; T, GSPE treatment effect; P, photoperiod effect; TxD, interaction between treatment and diet; DxP, interaction between diet and photoperiod; n.s., no significant differences. *, ** or *** Indicate significant differences by diet effect (*p* ≤ 0.05, *p* ≤ 0.01 and *p* ≤ 0.001, respectively), $ Indicate significant differences by treatment effect (*p* ≤ 0.05, respectively), #, ## or ### Indicate significant differences by photoperiod effect (*p* ≤ 0.05, *p* ≤ 0.01 and *p* ≤ 0.001, respectively) using 2-way and 3-way ANOVA followed by a LSD post-hoc test (*p* ≤ 0.05). & Indicates tendency using a LSD post-hoc test (*p* = 0.1–0.051). +, ++ or +++ Indicate significant differences using a paired Student’s *t*-test between ZT3 vs. ZT15 for each group (*p* ≤ 0.05, *p* ≤ 0.01 and *p* ≤ 0.001, respectively). x Indicates tendency using a paired Student’s *t*-test between ZT3 vs. ZT15 for each group (*p* = 0.1–0.051). AUC, Area under curve; GSPE, grape seed proanthocyanidin-rich extract, STD, Standard diet-fed rats; CAF, Cafeteria diet-fed rats; VH, rats administered vehicle; GSPE, rats administered 25 mg/kg GSPE; L12, normal photoperiod 12 h light per day; L18, long photoperiod 18 h light per day; L6, short photoperiod 6 h light per day. Grey background, dark phase. slash background, transitions phase.

A rhythm marked by the light/dark cycle was also detected by serum hormones. No differences by diet were found in the serum melatonin and corticosterone concentration (Figure 6A,B). Additionally, in both groups, a marked rhythm was observed depending on the light/dark phase with a higher concentration at ZT15 compared to ZT3. The CAF-fed rats showed an increase in serum testosterone concentration and the T3-to-T4 ratio at both ZT3 and ZT15 (Figure 6C,D). Furthermore, differences were observed between these two measurement points in both groups for both parameters, showing a rhythm marked by the light/dark cycle with a higher concentration at ZT3 with respect to ZT15 and in CAF rats compared to STD animals.

The change in the photoperiod had a significant light-hour-dependent effect on serum melatonin concentration (Figure 6E). Rats under L18 conditions showed no difference in melatonin between ZT3 and ZT15. However, when rats were transferred to the L6 photoperiod, the rhythm set by the light/dark cycle was remarkably maintained. Regarding corticosterone (Figure 6F), a photoperiod effect was found in CAF-fed rats that showed higher concentrations in L6 conditions at the ZT3 time point compared to L18 conditions (*p* = 0.031). This increase was mitigated by GSPE administration (*p* = 0.035). Interestingly, STD-fed rats also showed a lower concentration at ZT3 with GSPE administration under L18 conditions (*p* = 0.020). The rhythm set by the light/dark cycle was altered because of the increased concentration in CAF-VH rats at ZT3 under L6, while GSPE administration tended to restore this rhythm (*p* = 0.060). Serum testosterone concentration was also altered in a photoperiod-dependent manner (Figure 6G). Thus, the light/dark rhythm of the rats was lost under the L18 photoperiod. Additionally, CAF-fed rats in this photoperiod showed higher concentrations than STD-fed rats at both ZT3 and ZT15. At the ZT15 time point, GSPE administration reduced the hormone concentration (*p* = 0.010). When rats were under L6 conditions, they showed lower testosterone concentration at ZT15 compared to L18 photoperiod rats. Finally, the T3-to-T4 ratio showed no rhythm in CAF-fed rats at any photoperiod (Figure 6H). In addition, GSPE administration restored this rhythm in STD-fed rats at the L6 photoperiod.

## 4. Discussion

Circadian rhythm disruption has been related to behavior, metabolic, and physiological disorders [21]. This disturbance could be caused by changes in daylight or alterations to the normal light/dark cycle and may contribute to the development, jointly with a hypercaloric diet, of obesity and other metabolic syndrome comorbidities [21]. In addition, phenolic compounds have shown beneficial effects on several metabolic disorders, and recently some of these compounds, including PACs, have been related to the restoration of the circadian clock [28,34,35,36]. Therefore, the aim of our study was to evaluate the impact of an abrupt light/dark-cycle change on healthy and CAF-induced obese rats and if PACs were able to attenuate this photoperiod change effects. The GSPE dose used in this study was 25 mg/kg body weight, using a translation of animal to human doses [37] and estimating the daily intake for a 70 kg human, it corresponds to an intake of 370 mg/day.

As expected, the CAF diet produced a significant increase in BW gain compared to STD-fed rats. Because of the high fat and carbohydrate content, the CAF diet can induce a clinical picture of obesity and other comorbidities characteristic of metabolic syndrome [12,38]. Moreover, it was observed that CAF diet-fed rats presented hyperphagia since food and energy intakes reflected large difference between CAF- and STD-fed rats. Alterations to the photoperiod have also been related to an increase in BW [8,9,39]. Nevertheless, in this study, the sudden change in the light/dark cycle was not accompanied by body weight gain neither in STD nor CAF rats. These discrepancies could likely be due to the length of the study since we focused on the short-term effects of an altered photoperiod to avoid a potential adaptation of the animal to the new light/dark cycle (only one week). Therefore, a BW gain after a longer exposure time to the new photoperiod cannot be ruled out, since an adaptation of at least 5 days in the eating patterns after the reversion of the light–dark cycle has been reported [10]. In addition, the stress associated with a sudden change in the photoperiod could affect body weight [40,41]. Interestingly, it was observed that CAF-fed rats switched to the L6 photoperiod showed a greater loss of BW than their counterparts in the L18 photoperiod, demonstrating a diet–photoperiod interaction effect.

Regarding PACs effects, GSPE administration caused a BW loss in CAF-fed rats under L18 conditions, showing an interaction among photoperiod, diet, and GSPE since the same effect was not observed in the rest of groups. Several studies have reported lower BW gain after GSPE administration in obese animals, however, the rats were under L12 conditions, and the doses were higher than the 25 mg/kg used in this study [42,43]. Differences in GSPE bioavailability and bioactivity between healthy and diseased rats, including CAF-fed rats, has been previously reported by our group [44,45]. Related to the interaction between polyphenol consumption and photoperiods, it could be due to the different bioavailability of these compounds, depending on the circadian rhythm [46], and more specifically, on the exposure to daylight hours [47]. In this context, it has been reported that seasonal fruits rich in phenolic compounds, such as cherries, red grape, and oranges, showed photoperiod-dependent effects [36,48,49,50,51]. Regarding food intake, no differences in total energy intake were observed between the GSPE-treated CAF groups and CAF-fed VH groups after the photoperiod change. However, a change in feeding pattern was observed as GSPE administration increased fat intake compared to the corresponding CAF-fed VH groups. Moreover, this increase was photoperiod-dependent since it was significantly higher under the L18 than the L6 photoperiod. Disruption of the light/dark cycle combined with CAF diet has shown to increase the consumption of fat- and carbohydrate-rich foods in rats [52]. The results of this study showed that this effect appears to be stimulated by GSPE administration in a photoperiod-disturbance context.

As has previously been reported, CAF-fed rats under a L12 photoperiod showed higher EE than STD-fed rats [48]. The disruption to the light/dark cycle eliminated this difference between CAF-fed and STD-fed rats. A lower EE was reported in CAF-fed rats under exposure to the L18 or L6 photoperiods compared to the L12 photoperiod [53], which could explain in part the lack of differences in EE between CAF and STD rats after the photoperiod change. Interestingly, all groups showed differences in EE between light and dark phases, indicating that the rhythm marked by the light/dark cycle would not be altered by the sudden change in the photoperiod. In this context, GSPE administration did not show effects in EE in rats under the L6 photoperiod, whereas a decrease in EE was observed in CAF-fed rats administered GSPE, both in light and dark phases under L18 conditions. Although other studies reported an increase in EE after GSPE administration, the animals were under L12 conditions and without light/dark cycle disturbances [54,55]. The decrease in EE in the L18-CAF-GSPE group may be a result of the lower carbohydrate oxidation promoted by GSPE in CAF-fed rats under L18 conditions in both light and dark phases. GSPE has been shown to enhance fat oxidation, stimulating it in an obesogenic state [54,55], but no changes in carbohydrate oxidation have been reported with doses similar to 25 mg/kg [55]. Nevertheless, a photoperiod-dependent decrease in carbohydrate oxidation has been reported after cherry consumption [49].

Activity was also studied, and it was observed that CAF promoted sedentarism, since animals displayed less activity compared to the STD-fed rats both in light and dark phases. A decrease in activity has been reported in CAF-diet obese rats under L12 conditions and in rat jetlag and shiftwork models [38,39], but the mechanisms leading to this reduction are unclear. In this study, after the sudden change in the photoperiod, activity increased in STD-fed rats under L6 conditions from ZT6 to ZT12 compared to STD-fed rats under L18 conditions. This transition period was in light before the photoperiod change but in dark after the light/dark cycle change to L6. This increase in activity was mitigated by GSPE administration, thus maintaining the differences between light and dark phases. Interestingly, GSPE tended to increase activity in obese rats compared to VH rats under both photoperiods but only in the dark phase under the L6 photoperiod and from ZT12 to ZT18 under L18 conditions. This period was in dark before the photoperiod change but in light after the light/dark cycle disturbance.

The main marker of the light/dark cycle, melatonin, was not affected by the CAF diet under L12 conditions. This hormone increases its concentration during the dark phase, being an important signal for the organism to recognize the photoperiod to which it is exposed [56]. Thus, as expected, melatonin concentration showed a large difference between that measured at ZT3 and ZT15 under the L12 photoperiod both in the STD and CAF groups. However, after the switch of the photoperiod, changes in melatonin were observed since this hormone is the first to adapt to the new photoperiod [57,58,59], particularly under the L18 photoperiod, in which the light was switched off at ZT18. While melatonin signals to the organism to adapt its metabolism under changes in the photoperiod, corticosterone is another hormone with important metabolic implications. This hormone plays a key role in the hypothalamic–pituitary–adrenal axis (HPA) and presents a marked circadian rhythm [60,61]. Nevertheless, in this study, corticosterone did not adapt at the same rate as melatonin. An increase in corticosterone is related to stress [40,61] and its circadian disruption could lead to diseases of glucocorticoid sensitivity or resistance [62]. In this context, the increase in corticosterone levels observed in STD rats when they were switched to the L18 photoperiod, which were reversed by GSPE administration, could be due to the sudden change in the photoperiod and would relate to the increased stress of these animals. Interestingly, the CAF-VH group under the L6 photoperiod showed high corticosterone levels in the morning and did not follow their normal rhythm between ZT3 and ZT15. However, this effect was reversed by GSPE administration, recovering its rhythm. Therefore, GSPE could be attenuating the stress induced by the sudden change in the photoperiod. Hypothalamic–pituitary–gonadal (HPG) and hypothalamic–pituitary–thyroid (HPT) axes are important in processes such as lipid and carbohydrate metabolism and are closely related to biological rhythms. Testosterone and the T3-to-T4 ratio, respectively, act as modulators of these axes [63,64]. The rhythm of both hormones was lost due to the change in the photoperiod, which could lead to metabolic disturbances. Although GSPE administration reduced testosterone in L18 at ZT15 in CAF-fed rats, the rhythm was not recovered. In this sense, changes in testosterone levels by exposure to different photoperiods and by diet composition have been reported [65,66,67], a fact in agreement with our results showing a photoperiod-dependent variation and an increase by CAF diet. Moreover, as discussed above, the bioavailability of GSPE is different according to the photoperiod [47], modulating in a photoperiod-dependent manner the effect on the levels of this hormone. Regarding the T3-to-T4 ratio, the rhythm was lost in practically all groups, which could lead to metabolic disorders produced by changes in feeding patterns since this ratio has an established circadian rhythm dependent on diet [68,69].

## 5. Conclusions

In summary, the results suggested that grape seed PACs might be mitigating, in a photoperiod- and diet-dependent manner, the metabolic disturbances induced by an alteration to the light/dark cycle by suddenly changing the photoperiod. This photoperiod change could induce disorders in the HPG and HPT axes as their main modulators, such as testosterone and the T3-to-T4 ratio, respectively, lost the rhythm set by the light/dark cycle. Particularly, the T3-to-T4 ratio could also indicate an alteration to food intake and activity patterns since their circadian rhythm is closely linked to them. In addition, CAF-fed rats in the L6 photoperiod showed a significant increase in corticosterone at ZT3, causing a loss of rhythm for this hormone, closely linked to stress. GSPE administration was able to reduce serum corticosterone concentration, restoring its circadian rhythm, increase the T3-to-T4 ratio, and increase light phase activity in CAF-fed rats in the L6 photoperiod. In STD-fed rats, for this same photoperiod, the administration of PACs improved the testosterone circadian rhythm and T3-to-T4 ratio and reduced activity in the period which changed from the light to dark phase compared to the VH group. CAF-fed rats administered GSPE showed lower testosterone concentration, increased activity in the period which changed from the dark to light phase, and lost BW when switched to the L18 photoperiod. Finally, STD-fed rats in L18 showed a lower corticosterone concentration in the light phase after GSPE administration. All these different results showed how GSPE acted in a different manner depending on the photoperiod exposure and diet composition. Nevertheless, further studies are needed to elucidate the metabolic pathways and processes involved in these events.

## Figures and Tables

**Table 1:** Effect of an abrupt change in the photoperiod and grape seed proanthocyanidins administration on cumulative food and energy intake over the six weeks under L12 conditions and the seventh week of experiment under L18 and L6 conditions.

	Diet	L12 Cumulative 6 Weeks	*t*-Student	Photoperiod	VH 7th Week	GSPE 7th Week	ANOVA
Food Intake (g)	STD	120.21 (±3.41)	*p* < 0.001	L6	19.69 (±0.97)	22.61 (±1.44)	D
				L18	19.23 (±0.82)	20.05 (±0.80)	
	CAF	321.81 (±6.98)		L6	57.99 (±3.79) *	57.71 (±2.30) *	
				L18	48.93 (±5.64) *	51.94 (±4.61) *	
Energy Intake (Kcal)	STD	419.70 (±8.25)	*p* < 0.001	L6	65.76 (±3.24)	75.51 (±4.81)	D
				L18	64.22 (±2.74)	66.97 (±2.68)	
	CAF	1335.17 (±28.45)		L6	233.08 (±18.34) *	233.64 (±10.32) *	
				L18	192.59 (±31.13) *	216.21 (±27.48) *	
Energy intake from Protein (Kcal)	STD	79.74 (±1.57)	*p* < 0.001	L6	12.50 (±0.62)	14.35 (±0.91) ^&^	D
				L18	12.20 (±0.52)	12.72 (±0.51)	
	CAF	119.62 (±1.38)		L6	16.06 (±0.76) *	17.27 (±0.43) *	
				L18	16.52 (±0.65) *	17.78 (±1.33) *	
Energy intake from Carbohydrates (Kcal)	STD	302.19 (±5.94)	*p* < 0.001	L6	47.35 (±2.33)	54.36 (±3.46)	D, P
				L18	46.24 (±1.97)	48.22 (±1.93)	
	CAF	911.50 (±28.22)		L6	179.12 (±15.87) *	172.16 (±10.30) *	
				L18	138.59 (±27.29) *^,#^	145.83 (±26.77) *	
Energy intake from Fat (Kcal)	STD	33.58 (±0.66)	*p* < 0.001	L6	5.26 (±0.26)	6.04 (±0.38)	D, T, DxT, DxP
				L18	5.14 (±0.22)	5.36 (±0.21)	
	CAF	416.76 (±7.35)		L6	50.04 (±3.57) *	57.08 (±1.23) *^,$^	
				L18	52.80 (±3.76) *	66.81 (±4.67) *^,$,#^	
AUC Energy from carbohydrates oxidation	STD	75,639.10 (±9703.77)	*p* = 0.02	L6	100,366 (±21,217)	112,676 (±11,496)	D
				L18	112,095 (±11,294)	106,221 (±5877)	
	CAF	49,412.4 (±6075.05)		L6	71,406 (±9041) *	67,067 (±14,008) *	
				L18	69,592 (±6221) *	35,767 (±11,233) *^,$^	
AUC Energy from fat oxidation	STD	111,461 (±11,654)	*p* = 0.008	L6	64,388 (±16,072)	48,017 (±11,396)	D
				L18	50,167 (±11,689)	48,442 (±2447)	
	CAF	165,171 (±15,493)		L6	97,490 (±9362) *	94,484 (±16,432) *	
				L18	130,001 (±22,824) *	122,074 (±16,528) *	

Values are expressed as the mean ± S.E.M. (*n* = 24) for L12 conditions. *P*-value was calculated using a Student’s *t*-test between L12-STD vs. L12-CAF (*p* ≤ 0.05). Values are expressed as the mean ± S.E.M. (*n* = 6) for L18 and L6 conditions. D, diet effect; T, GSPE treatment effect; *p*, photoperiod effect; TxD, interaction between treatment and diet; DxP, interaction between diet and photoperiod. * Indicates significant differences by diet effect, $ Indicates significant differences by treatment effect, # Indicates significant differences by photoperiod effect using 3-way ANOVA followed by a LSD post-hoc test (*p* ≤ 0.05). & Indicates tendency using a LSD post-hoc test (*p* = 0.1–0.051). AUC, Area under curve; STD, Standard diet-fed rats; CAF, Cafeteria diet-fed rats; VH, rats administered vehicle; GSPE, rats administered 25 mg/kg grape seed proanthocyanidin-rich extract; L12, standard photoperiod 12 h light per day; L18, long photoperiod 18 h light per day; L6, short photoperiod 6 h light per day.

## Data Availability

The data presented in this study are available on request from the corresponding author. The data are not publicly available due to lack of platform to publish it.

## Supplementary Materials

The following are available online at https://www.mdpi.com/article/10.3390/nu14091834/s1, Table S1: Statistical summary.
