# Supplementary material for: Grape Seed Proanthocyanidins Mitigate the Disturbances Caused by an Abrupt Photoperiod Change in Healthy and Obese Rats
Source: Nutrients. 2022 Apr 27;14(9):1834. doi: 10.3390/nu14091834 (PMC9100649; doi:10.3390/nu14091834)
Supplement: Supplementary file 1 [file nutrients-14-01834-s001.zip › nutrients-1692315-supplementary.pdf]

Table S1. Statistical summary.

Parameter	2-way ANOVA for L18 conditons	2-way ANOVA for L6 conditons	3-way ANOVA
Body weight gain in 7th week	DxT	n.s.	TxD
Energy intake in 7th week	D	D	D
AUC Energy Expenditure light phase	T	n.s.	T, P
AUC Energy Expenditure dark phase	n.s.	D	T, D
AUC Energy from carbohydrate Oxidation light phase	D	n.s.	D
AUC Energy from carbohydrate Oxidation dark phase	D	D	D
AUC Energy from fat Oxidation light phase	D	n.s.	D
AUC Energy from fat Oxidation dark phase	D	D	D
AUC Vertical activity light phase	D	D	P, D, TxP
AUC Vertical activity dark phase	D	D	D, P
Melatonin light phase	D	D	D
Melatonin dark phase	n.s.	n.s.	P
Corticosterone light phase	T	n.s.	T
Corticosterone dark phase	n.s.	n.s.	P
Testosterone light phase	D	n.s.	P
Testosterone dark phase	TxD	D	D, P, TxD
T3/T4 light phase	n.s.	n.s.	n.s.
T3/T4 dark phase	n.s.	D	D

D, diet effect; T, GSPE treatment effect; P, photoperiod effect; TxD, interaction between treatment and Diet; TxP, interaction between GSPE treatment and Photoperiod.
